# Perceptions of Risk Stratification Workflows in Primary Care

**DOI:** 10.3390/healthcare5040078

**Published:** 2017-10-21

**Authors:** Rachel L. Ross, Bhavaya Sachdeva, Jesse Wagner, Katrina Ramsey, David A. Dorr

**Affiliations:** Department of Medical Informatics & Clinical Epidemiology, Oregon Health & Science University, Portland, OR 97239-3098, USA; sachdeva@ohsu.edu (B.S.); wagnerje@ohsu.edu (J.W.); ramseyk@ohsu.edu (K.R.); dorrd@ohsu.edu (D.D.)

**Keywords:** risk stratification, chronic care, primary care, forecasting, care management, risk assessment

## Abstract

Risk stratification (RS) in primary care is frequently used by policy-makers, payers, and health systems; the process requires risk assessment for adverse health outcomes across a population to assign patients into risk tiers and allow care management (CM) resources to be targeted effectively. Our objective was to understand the approach to and perception of RS in primary care practices. An online survey was developed, tested, and administered to 148 representatives of 37 primary care practices engaged in RS varying in size, location and ownership. The survey assessed practices’ approach to, perception of, and confidence in RS, and its effect on subsequent CM activities. We examined psychometric properties of the survey to determine validity and conducted chi-square analyses to determine the association between practice characteristics and confidence and agreement with risk scores. The survey yielded a 68% response rate (100 respondents). Overall, participants felt moderately confident in their risk scores (range 41–53.8%), and moderately to highly confident in their subsequent CM workflows (range 46–68%). Respondents from small and independent practices were more likely to have higher confidence and agreement with their RS approaches and scores (*p* < 0.01). Confidence levels were highest, however, when practices incorporated human review into their RS processes (*p* < 0.05). This trend was not affected by respondents’ professional roles. Additional work from a broad mixed-methods effort will add to our understanding of RS implementation processes and outcomes.

## 1. Introduction

Risk stratification (RS) is a population health management process through which empaneled patients are evaluated for risk of poor health outcomes by an algorithm (computer-generated or manual), human review, or combination of both approaches. In the context of this paper, “human review” is the overarching term referring to a risk stratification approach that involves either adjudication (clinical review of a previously calculated score) or pure clinical intuition (relying solely on clinical gestalt to assign a score). Regardless of the type of approach, patients are typically assigned a risk score or placed into a risk group based on diagnoses, healthcare utilization, social and behavioral risks, and other factors that may impact future health status. The ultimate goal of RS is to reduce adverse health outcomes that drive overutilization of healthcare services and increased costs via proactive, intensive care management (CM) of patients deemed “high-risk”. Various risk-adjustment models demonstrate successful identification of these high-cost, high-needs patients [1]. Upon selection of high-risk patients, intensive management approaches (e.g., behavioral health models, social service programs, or care coordination) can be targeted to address the needs of these vulnerable high-cost, high-needs patients. An estimated 5–20% of individuals in a given primary care practice can see improved outcomes as a result of such interventions; this is of critical importance because this portion of the patient population is responsible for over half of our country’s total health care costs [2]. 

Given its potential to inform targeted CM and thereby reduce costs and healthcare utilization, RS has become a key provision of contemporary primary care transformation efforts such as the Comprehensive Primary Care (CPC) initiative, its expansion program, CPC Plus, Patient-Centered Medical Home models, the use of Chronic Care Management codes, and many Accountable Care Organization and other global payment initiatives. With this increased emphasis on RS, practices need to understand applied methodologies and their associated benefits and hurdles; RS is the number one reported issue for health systems improving use of data for health reform [3]. Despite recognizing its need in primary care and exploring existing methodologies for almost a decade [4], there is a relative dearth of evidence regarding how primary care practices implement RS workflows. Many studies have examined condition-specific RS in hospital settings [5,6,7], analyzed the predictive validity of specific algorithms [8,9], and investigated the ethical ramifications of categorizing patients into risk tiers [10], yet few have actually assessed the impact and challenges of implementing RS among all patients in a primary care environment. The lack of guidelines and literature on RS in primary care can create a challenge for practices hoping to implement such workflows. In a primary care health reform initiative led by the Centers for Medicare & Medicaid Services, only 14% of the 500 practices had any substantial experience in RS [11,12]. Thus, most practices appear to be diving into this challenging and multi-faceted process without much structure or guidance.

Implementing RS requires mapping an algorithm to extant data, validating the results, identifying segments of patients at high-risk, and then integrating the results into population management approaches [13]. Many primary care practices lack the technical expertise to map data and validate it [14], leading to significant concerns about accuracy and usability. However, the technical concerns are not the sole challenge. Evidence suggests that practices may initially adopt an approach that is not fully supported or understood, and later tailor it to better match their clinical perception of patient risk. One such qualitative study revealed that even an externally provided score required significant time to resolve conflicting perceptions, and most providers expressed that the tested approach ‘raised many questions’ [15].

Given these challenges and the increasing emphasis on risk-stratified CM in primary care (due its potential to reduce cost and utilization among high-needs patients), our study aims to (a) assess primary care practices’ perceptions of their RS and CM approaches, and (b) identify factors that lead to increased confidence in RS implementation and its successful use in CM interventions. We hypothesized that RS methods involving human review would be associated with improved perceptions of RS and CM workflows, to account for the value of clinical input and the common HIT-related challenges in primary care. Furthermore, we predicted that factors pertaining to respondents’ perceptions of RS would be correlated with their perceptions of CM outcomes, and anticipated that this trend would be influenced by respondents’ roles within the practice, as well as with various practice demographics. Survey results were compared across practice size, location, and ownership, as these variables have been shown to affect the ways in which a practice addresses the needs of their population and delivers care [16,17,18]. This survey is part of a larger mixed-methods study aiming to identify the most efficient and effective primary care RS implementation methods. 

## 2. Materials and Methods 

The study team developed the inceptive survey to gather information from the following domains: (a) practice demographics and characteristics, (b) RS approach—Implementation and methodology, (c) perception and effects of RS implementation, (d) perception of CM activities and performance, and (e) reflection on lessons learned and needs for improving RS. The study team found no pre-existing survey inclusive of these topics. In-depth reviews of the existing literature, as well as feedback from experts in the field and practice representatives, determined the survey questions, with seven iterative versions created. Experts included researchers who had published qualitative or quantitative papers on risk stratification, or had presented on institutional approaches for risk stratification. These individuals reviewed the initial question sets until consensus was achieved on the clarity and completeness of the survey for the domains. Next, the survey was tested by five representatives from a small group of practices (n = 3) to assess feasibility and elicit feedback. The practice representatives were observed completing the survey, and asked to comment on clarity, relevance, and meaning of the questions. All feedback was taken into account for revisions, which were presented back until consensus was reached. 

The survey asked respondents to enter information regarding practice demographics (multiple-choice questions), professional role (free-response), RS approaches (multiple-choice), and perceptions of RS and CM approaches (5- or 9-point Likert scale). To address perceptions of RS and CM, practices were asked to rate their confidence in their RS methodologies, and agreement with their scores and subsequent ability to effectively target patients to receive care management resources. For construct validity, we completed factor analyses on the 4 RS perception questions, the 5 CM perception questions, and then the 9 questions together, all using Varimax rotation. This allowed us to generate normalized composite scores for RS and CM perceptions and examine inter-item correlations and Cronbach’s alpha using SAS software version 9.4. RS and CM constructs were tested separately. The analyses demonstrated that the RS and CM perception question sets had strong evidence to be two individual constructs with scores between 0.8 and 0.9.

The administration of the survey was conducted in accordance with the study protocol approved by the Institutional Review Board at Oregon Health and Science University under protocol number 8277. We identified practices participating in the Comprehensive Primary Care (CPC) initiative, a national healthcare transformation model requiring RS. From this initial set of practices, we targeted a subset representing a broad range of sizes (large, medium, or small), ownership (independent, health-system-operated, or other), and location (urban, suburban, or rural). Practices engaged in RS from Oregon, Colorado, Ohio, and Kentucky were invited to participate in the study. We recruited at least one respondent from each participating practice whose role included the creation or use of risk scores (typically a practice manager or care manager), and used a snowball technique to further identify additional respondents from the practice. Once practices agreed to participate, the practice representative(s) provided email addresses for the identified survey recipients. We intentionally engaged participants who were knowledgeable about RS use to generate higher degrees of accuracy for questions pertaining to RS workflows and effects. The survey was administered using Research Electronic Data Capture (REDCap), managed by the Oregon Clinical and Translational Research Institute (OCTRI) and OHSU and developed at Vanderbilt [19].

Descriptive statistics were applied to understand the sample at both the practice and individual respondent level. Pearson’s Correlation and chi-square tests were calculated to determine if practice characteristics (size, location, and ownership) were associated with RS approach, confidence, and score agreement; scores of 1–3 were classified as disagreement with RS/CM process success, 4–6 as neutral perception of process success, and 7–9 as agreement with process success. We compared group differences in mean composite RS and CM perceptions using separate linear regression models with algorithm type (computer, human, or both) and clinic role (physician, care coordinator/manager, administrator, and staff/other) as independent variables.

## 3. Results

### 3.1. Survey Respondent Characteristics

In all, 100 of 148 survey recipients responded (68% response rate), representing 37 practices, resulting in an average of 2.7 responses per practice (range 1–12 respondents per practice). Approximately half (51%) of practices had more than one respondent, and of the practices who had multiple respondents, 32% (n = 6) had significant variation in their perception of their RS process (SD > 0.2). Submission of a completed or partially completed survey constituted a response; none of the participants submitted a survey with <50% completion. Table 1 demonstrates the basic breakdown of practice-reported characteristics and individual practice roles. The majority of respondents were care coordinators or care managers, followed by those in administrative/managerial roles, followed by providers. This information suggests that these roles represent the staff members most directly involved in developing and utilizing RS in the practice setting, given that the practice was responsible for identifying the most appropriate individuals to complete the survey. 

As expected, given our selection of practices from an initiative incentivizing RS, Table 2 shows that respondents had significant experience in the RS process. The type of RS used varied widely; in all, 48% reported using a computer algorithm to generate their scores, and 83% incorporated human review to either (a) generate the score purely from gestalt, or (b) “adjudicate”, or alter a computer-generated algorithm. Thus, most clinics are completing RS assessments manually or semi-manually through an adjudicated approach. The majority of participants used their own reports or systems to generate RS data, including EHR registry systems. Additionally, most respondents reported adjusting their RS methodology at some point since initial implementation.

### 3.2. Perception of RS and CM

**Figure 1:**
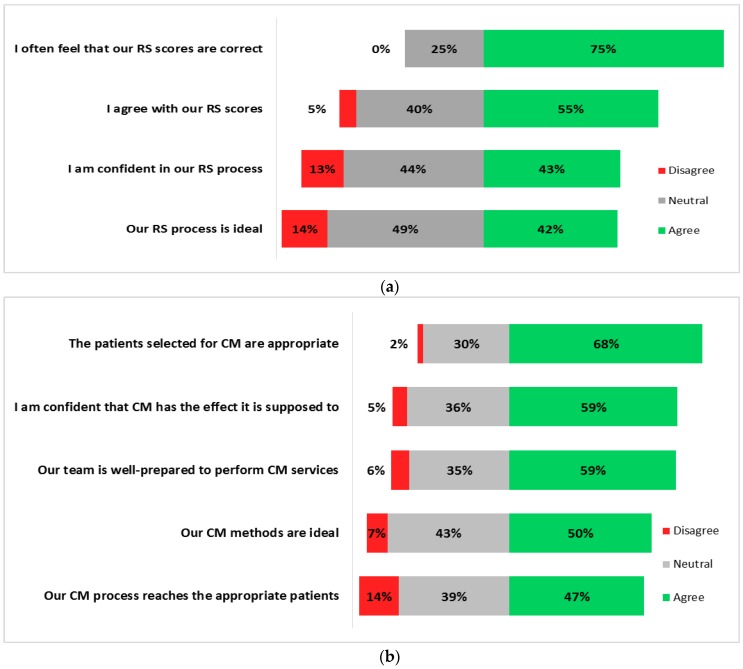
Individual perceptions of (**a**) risk stratification outcomes, and (**b**) care management outcomes.

The survey asked about participants’ sense of how well the process worked for RS and for CM. Likert Scale responses from 1 to 3 were classified as “disagreement”, 4–6 were classified as “neutral” or “moderate” agreement, and 7–9 were classified as “high agreement”. Participants had moderate agreement (Figure 1a, range 41–53.8%) with the statements about their RS process and score outcomes in terms of agreement, confidence, and whether the process was ideal. Participants also showed moderate to high agreement (Figure 1b, range 46–68%) with statements about their CM experience. The highest agreement (68%) was to the statement, “The patients selected for CM are appropriate”, while the lowest was to, “Our CM process reaches the appropriate patients”. This response indicates that selecting the appropriate patients may be easier than actually reaching them.

Three factor analyses were completed. With only CM questions, 70% of variance was explained in a single factor. For RS questions only, the factor analysis revealed one factor with 75% of variance explained. We also completed a factor analysis with both RS and CM to see if all responses were the same; two factors emerged on the constructs, both with Eigenvalues >1, and the Cronbach’s alpha values of the two constructs were 0.87 and 0.89, respectively. These constructs were then used to compare overall perceptions of RS and CM to other clinic characteristics.

**Figure 2:**
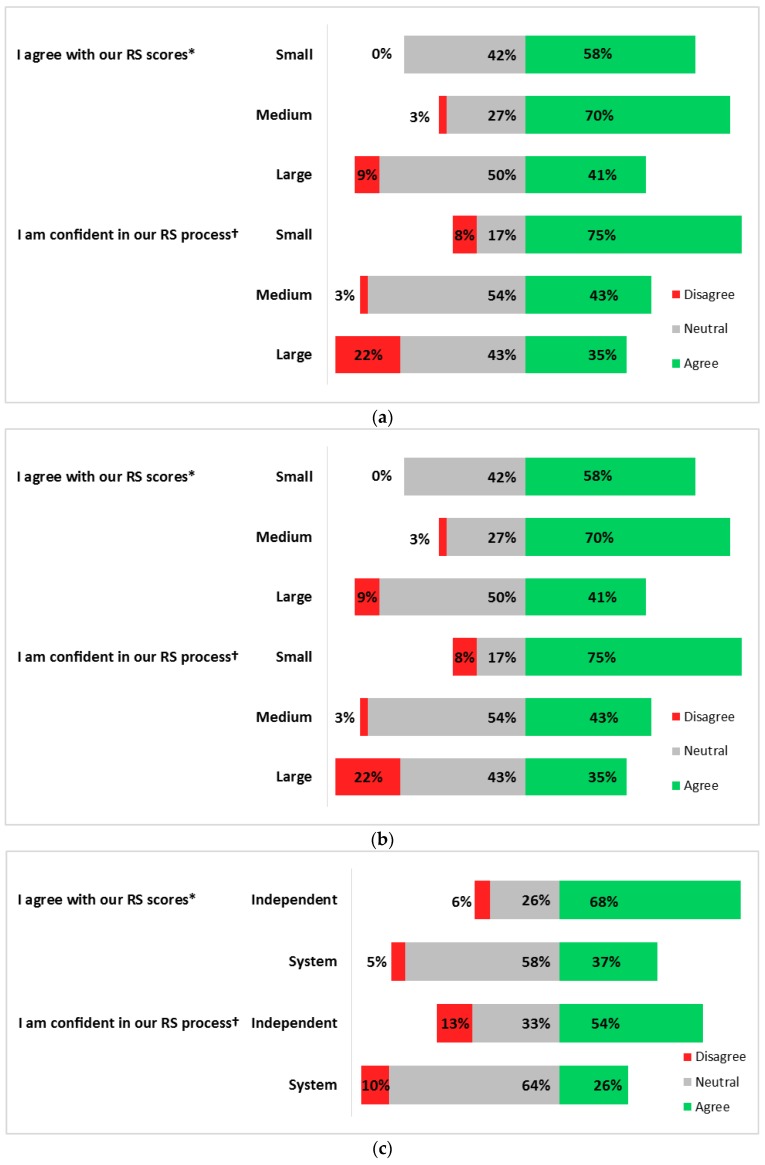
Risk stratification confidence and agreement by (**a**) Practice size (* χ^2^ = 7.0, *p* = 0.030; ^†^ χ^2^ = 6.4, *p* = 0.041); (**b**) Practice location (* χ^2^ = 4.4, *p* = 0.112; ^†^ χ^2^ = 2.1, *p* = 0.344); and (**c**) Practice ownership (* χ^2^ = 8.5, *p* = 0.004; ^†^ χ^2^ = 7.3, *p* < 0.001).

Within the RS construct, we found that several practice characteristics related to confidence and agreement scores (Figure 2a–c). Fifty-eight percent of small practice representatives felt confident in their risk scores (size: confidence χ^2^ *p* < 0.01), and 75% felt confident in their RS methods (size: agreement χ^2^ *p* < 0.01), while 68% of representatives from independent practices felt confident in their scores and 54% felt confident in their methods (χ^2^ *p* < 0.001).

### 3.3. Participant Experience

Inclusion of human review was associated with significantly higher confidence in the RS process (Table 3). Composite mean RS ratings were lowest for computer-only algorithms (4.9 on a scale of 1 to 9); mean ratings for human review alone were nearly a full standard deviation (SD) higher (+1.6), and a half SD higher than the combination of computer and human review (+0.9). Care managers and care coordinators had the highest confidence in the RS process by a small margin, averaging 6.2, +0.3 from staff and physicians, who tied as the lowest categories. Similarly, the use of clinical intuition in RS was associated with greater confidence in subsequent CM approaches (mean score 7.0), though the correlation between the two composites was only 0.26 (*p* = 0.01), and respondents with computer-only algorithms rated their CM process higher than computer and human intuition combined (6.5 vs. 6.0, respectively). Physicians generated the highest confidence ratings in their CM workflows, while care managers and care coordinators, who tended to have higher satisfaction with their RS process, had the lowest confidence in their CM approach across all algorithm types, but these effects were non-significant (*p* = 0.4).

## 4. Discussion

Given the increasing emphasis on risk-stratified CM in contemporary healthcare improvement work, the complexity of approaching this workflow, and the lack of evidence that exists to support and understand facilitation of RS in primary care, research on RS implementation is critical. The application of this population-management technique in primary care is still nascent, and thus, we must analyze and understand the experiences of the first several waves of adopters. The current study brings to light the experiences of those in primary care actively working to develop and refine their RS approaches.

Previous literature shows primary care staff often face difficulties incorporating new metrics, and that gaps remain between the practice and theory behind successful implementation [20]. Participants in the present study appeared to have mixed feelings about RS implementation and CM processes, but tended to feel slightly more confident overall about CM than RS. Examining these constructs in tandem is necessitated by the cyclical nature of the RS processes’ downstream effects on the CM processes, and subsequent effect of the CM outcomes’ potential effects on the perceptions of the RS processes. This finding indicates that further investigation is needed into practices’ processes for tailoring CM activities, to discern the workflow elements in the CM process that result in greater confidence in predicting high-needs patients. 

The data demonstrate that the vast majority of study participants included human review in their approach. Further analyses revealed that people tend to feel most positive about the process when human review is incorporated. More specifically, human review approaches generated the highest levels of confidence. Individuals in all role categories displayed this same pattern. Those in care coordination and care management roles were the most likely to feel confident in their RS approach; this is perhaps due to the increased likelihood that these individuals are involved in both the creation and utilization of the processes. Furthermore, small and independent practices tended to feel more confident in their RS processes. This finding can be attributed to a number of factors: (a) these practices are likely to experience greater ease in communication between and within departments, (b) the process is less likely to be centralized by a separate division within an organization, and (c) the combination of these dynamics may lead to enhanced ability for process or algorithm refinement by care team members. This finding should be taken into consideration by practices who are in the process of planning or refining their RS approach; larger or system-owned practices may benefit from developing RS processes on smaller scales within individual practices, or including practice-level representatives in communications about risk score implementation processes. Further analysis is needed to assess the feasibility of this work.

Since RS is still relatively new at the primary care level, the practices involved in RS and in the survey are likely to be somewhat different from others, which may affect the generalizability of these findings. Practices were selected to participate based on their experience implementing RS through participation in a larger healthcare initiative involving monthly payments for caring for a population. Currently, nearly all primary care practices receive similar fees—whether through similar transformation programs, through risk-tiered Chronic Care Management fee-for-service codes, or as part of global budgeting. We involved practices from three states to limit local bias. 

Limitations of this work encompass several potential factors. While analyses show independent and small practices as more likely to be confident and agree with their RS process and scores, small practices were underrepresented, accounting for 16% of our total sample size. Within participating practices, people involved in RS varied, which may reduce the likelihood of statistically significant distinctions between roles and RS perception; survey respondents were intimately involved in the process, and may have more positive or more negative feelings than those not involved in the same role. We did not have the power to explore intraclinic variation through modeling or interviews, which may be an important factor in itself. Additionally, significant staff turnover may have limited some respondents’ knowledge and experience with RS, which could affect RS and CM perception responses. Finally, the survey did not go through complete validation, especially for predictive or concurrent validity (e.g., do their perceptions relate to more concrete measures of RS and CM success).

As RS spreads in primary care and elsewhere, larger, more representative samples should be assessed to understand the initial perception and change over time of those perceptions. For example, more than two-thirds of respondents had changed their RS approach at least once. Further research is warranted to ascertain the commonality of this occurrence, and to determine the number of iterations necessary to refine an ideal approach. Additionally, Hong et al. reported that training provided on how to stratify risk yielded improved predictions of downstream risk compared to the initial approach [21].

More information is needed about the finding that few practices chose to use computer algorithms without any human review. It is unclear if this is due to the fact that, given the choice, people are inherently less confident in computer algorithms (lacking the opportunity to incorporate human decision-making), if practices are less likely to be able to implement complex algorithms pulling from their existing data sources, or if this finding is due to another reason entirely.

The present study is part of a larger mixed-methods project to analyze successful primary care RS implementation. Future qualitative and quantitative analyses will look at whether variations in approach and perception did identify more patients at high risk, and what additional barriers exist to improving perceptions of the approach.

## 5. Conclusions

We successfully created, partially validated, and administered a survey on RS, with significant variability on perceptions of RS and CM processes. The results highlight the importance of the human element in this complex process, which should be considered for future RS programs at the point-of-care.

## Figures and Tables

**Table 1:** Practice-level and participant-level characteristics of respondents.

**Practice-Level Responses**		**Counts (n = 37) n (%)**
Size ^1^	Small	6 (16%)
	Medium	11 (30%)
	Large	20 (54%)
Ownership	Independent	13 (35%)
	Part of IPA	8 (22%)
	Health System	15 (41%)
	Other	1 (3%)
Location	Urban	20 (54%)
	Suburban	7 (19%)
	Rural/Frontier	10 (27%)
**Participant-level responses**		**Counts (n = 100) n (%)**
Roles	Care Coordinator/Manager	47 (47%)
	Administrator/Manager	21 (21%)
	Physician	15 (15%)
	Staff/Other	16 (16%)

^1^ Small practices are defined as having <3000 active patients, medium practices are defined as having between 3000 and 8000 active patients, and large practices are defined as having >8000 active patients.

**Table 2:** Participant experience with risk-stratification workflows.

Characteristics of RS	Mean (Median) (Range)
% of Active Population Risk Stratified	77 (90) (0–100)
Duration of Risk Stratification Activities (months)	22.9 (22.9) (12–106.9)
**Type of RS**	**Count of “Yes” n (%)**
Computer-generated algorithm	48 (48%)
If Computer Algorithm, Simple Algorithm	11 (23%)
…, Moderate Complexity Algorithm	25 (52%)
…, Complex Algorithm	9 (19%)
Human Review	83 (83%)
If Human Review, Adjudication of algorithm	37 (45%)
…, Clinical Intuition only	35 (42%)
**Technology Used in RS Process**	**Count of Responses n (%)**
EHR/Registry Reports	81 (81%)
Payer/Third Party Reports	37 (37%)
EHR System w/general ambulatory or specific CM tools	61 (61%)
Interactive Population Management System	21 (21%)
**Change in RS Method During Implementation**	**Count of Responses n (%)**
Yes	62 (62%)
No	35 (35%)

**Table 3:** Participant experience with risk-stratification workflow.

	Risk Score Perception Combined Score (1–9 Scale)				Care Management Perception Combined Score (1–9 Scale)			
Overall mean ± SD	6.0 ± 1.7				6.6 ± 1.5			
	Mean	Diff *	95% CI ^†^	*p* (adj) ^‡^	Mean	Diff *	95% CI	*p* (adj) ^‡^
**Score type**								
Computer alone	4.9	(ref) *	—	0.002	6.5	(ref)	—	0.02
Computer + Adjudication	5.8	0.9	[−0.3, 2.0]		6.0	−0.4	[−1.6, 0.7]	
Human review alone	6.5	1.6	[0.5,2.8]		7.0	0.5	[−0.7, 1.7]	
**Clinic role**								
Staff/Other	5.8	(ref)	—	0.64	6.8	(ref)	—	0.71
Physician	5.8	0.02	[−1.2, 1.3]		7.0	0.2	[−1.1, 1.4]	
Admin/Manager	6.0	0.2	[−0.9, 1.3]		6.5	−0.3	[−1.4, 0.8]	
Care Coordinator/Manager	6.2	0.3	[−0.6, 1.3]		6.3	−0.5	[−1.5, 0.5]	

* Diff: Difference in mean from reference (ref) category; ^†^ 95% CI: 95% confidence interval for the difference; ^‡^ *p*: *p*-value from global test of effect, before and (after) adjusting for clinic effect with cluster-robust standard errors.

## Supplementary Materials

The Risk Stratification and Care Management Survey will be available online at www.mdpi.com/2227-9032/5/4/78/s1.
