# Supplementary material for: Perceptions of Risk Stratification Workflows in Primary Care
Source: Healthcare (Basel). 2017 Oct 21;5(4):78. doi: 10.3390/healthcare5040078 (PMC5746712; doi:10.3390/healthcare5040078)
Supplement: Supplementary file 1 [file healthcare-05-00078-s001.pdf]

Risk Stratification + Care Management Survey

This survey should be filled out on-line by those persons most connected to the development process and evaluation of the overall risk stratification process in the clinic.

This survey will ask about the use of risk stratification and care management in your clinic. Please answer the following questions to the best of your knowledge. Your responses will be kept confidential.

Your clinic role: ______________________

Other clinic roles related to risk stratification and care management: ______________________

Clinic size:
- ◯ Small (less than 3,000 patients)
- ◯ Medium (3,000-8,000 patients)
- ◯ Large (greater than 8,000 patients)

Clinic location:
- ◯ Urban
- ◯ Suburban
- ◯ Rural
- ◯ Frontier (located more than 60 miles from a town of pop. 20,000)

Clinic ownership:
- ◯ Independent
- ◯ Part of an Independent Practice Association
- ◯ Other quasi-independent structure
- ◯ Part of health system
- ◯ Other

If "other," please specify: ______________________

Specialty mix:
- ☐ Family medicine
- ☐ Internal medicine
- ☐ Pediatrics
- ☐ Women's health
- ☐ Other specialties

If "other," please describe: ______________________

Briefly describe the current status of efforts to incorporate risk stratification into your practice's routine ______________________

What percent of your active patients are risk stratified? ______________________
(Enter number without percent (%) symbol)

Approximately when did you implement your risk stratification method? (Month/Day/Year) ______________________

About how long did it take to initially implement risk stratification?
- ◯ 1-3 months
- ◯ 4-6 Months
- ◯ 7-9 months
- ◯ 10-12 months
- ◯ more than a year

Has the risk stratification method changed over time?
- ◯ Yes
- ◯ No

If yes, please briefly explain: ______________________

The next two questions will inquire about your current risk stratification method. The first asks about computer-generated algorithms, and the second about human review.

Do you use a computer-generated algorithm in your risk stratification process?
- ◯ Yes
- ◯ No

How would you describe your current approach (select all that apply):
- ☐ Simple algorithm (chronic illnesses)
- ☐ Moderately complex algorithm (2+ sources of data, but relatively simple to understand)
- ☐ Very complex algorithm (weighting, integration of much different data, etc)
- ☐ Other (please explain)

If "other," please explain: ________________________________

What specific scoring method do you use, if any (select all that apply)?
- ☐ HCC scoring
- ☐ Chronic Disease Payment System (CDPS)
- ☐ ACA qualified conditions
- ☐ A Charlson Comorbidity Score
- ☐ The AAFP approach
- ☐ AGC / DxGC
- ☐ None
- ☐ Other (please explain)

If "none" or "other," please explain: ________________________________

Do you use human review to provide or adjust risk scores in your process?
- ◯ Yes
- ◯ No

If yes, how? (please check all that apply)
- ☐ To adjudicate computer-scores (verify or change the computer assessed scores)
- ☐ To generate a risk score by itself (clinician or staff rates them high risk on intuition alone)
- ☐ Other (please explain)

If "other," please explain: ________________________________

Please briefly describe what you believe is the purpose of risk stratification. ________________________________

Please rate the following statements based on how true they are to your own experiences with risk stratification implementation, on a scale of one to nine: 1 - Completely disagree, 9 - Completely Agree (or n/a if not applicable)

	Completely disagree 1	2	3	4	Neutral 5	6	7	8	Completely agree 9	n/a
Our risk stratification method is ideal.	◯	◯	◯	◯	◯	◯	◯	◯	◯	◯
I am confident that the risk stratification scores predict what they are supposed to - future utilization, worsening health problems, or similar issues.	◯	◯	◯	◯	◯	◯	◯	◯	◯	◯
I agree with the risk stratification scores after the process is complete.	◯	◯	◯	◯	◯	◯	◯	◯	◯	◯

How often do you feel the risk stratification scores are correct?

◯ Never
◯ Rarely
◯ Sometimes
◯ Most of the time
◯ Nearly always

Who (personal or organizational position) was most helpful in implementing risk stratification? Why?

____

Going forward, what 3 things do you need in order to continue (or start) to more effectively use risk stratification?

1. ______________________________

2. ______________________________

3. ______________________________

What is the most important lesson you have learned regarding the implementation of risk stratification?

____

- Care Management -

The following questions are related to how care management is incorporated into your clinic.

In your clinic, approximately how many people do care management? ______________________________

On average, what percent of their time is dedicated to care management? ______________________________
(Enter number without percent (%) symbol)

What are the professional designations of these FTE who are specifically assigned to care management (select ALL that apply)?

- ☐ Nurse (RN, LPN, LVN)
- ☐ Social worker
- ☐ Behaviorist
- ☐ Medical assistant / nursing assistant
- ☐ Mid-level provider (NP, PA)
- ☐ Provider - MD, DO, etc
- ☐ Pharm D
- ☐ Other (indicate below)

Other provider: __________________________________

What training do they have in care management (select all that apply)?

- ☐ Certification for Care Management
- ☐ Took a course
- ☐ On the job training

What incentives do you receive/provide for care management? (select all that apply)

- ☐ Shared savings
- ☐ Per member per month fee
- ☐ None
- ☐ Other (please explain)

If "other," please explain: __________________________________

Do these performance awards go to individuals or are they calculated as team awards?

- ◯ Individual
- ◯ Team

What technology do you use for care management and/or risk stratification (select all that apply)?

- ☐ Reports from EHR or registry system
- ☐ Reports from payers or third parties
- ☐ EHR system with specific CM tools
- ☐ EHR system with general ambulatory tools
- ☐ Interactive population management system (can select / filter reports)

What care management activities do your care managers engage in (select all that apply)?

- ☐ Monitoring (frequency)
- ☐ Scheduled phone visits
- ☐ Scheduled home visits
- ☐ Warm hand-offs in the office
- ☐ Pre-visit planning
- ☐ Post-visit followup
- ☐ Referral tracking and follow-up
- ☐ Hospital follow-up
- ☐ ED follow-up
- ☐ Care Coordination - with specialist or community resources
- ☐ Health Coach/Motivational Interviewing
- ☐ Disease specific education
- ☐ Goal setting
- ☐ Other (please explain)

If 'other' please explain: __________________________________

Please rate the following statements based on how true they are to your own experiences with care management, on a scale of one to nine: 1 - Completely disagree, 9 - Completely Agree (or n/a if not applicable)

	Completely Disagree 1	2	3	4	Neutral 5	6	7	8	Completely Agree 9	n/a
Our care management methods are ideal.	○	○	○	○	○	○	○	○	○	○
I am confident that care management has the effect it is supposed to in our clinic - mitigate risk, help patients meet their goals, and keep them healthier.	○	○	○	○	○	○	○	○	○	○
The patients selected for care management are appropriate for the interventions.	○	○	○	○	○	○	○	○	○	○
Our team is well-prepared to perform care management services.	○	○	○	○	○	○	○	○	○	○
We are able to reach a substantial proportion of our patients who would benefit from care management.	○	○	○	○	○	○	○	○	○	○

Thank you for taking the time to complete our survey. Your feedback is valuable. Please use the space below if you have any additional comments or general feedback.

Additional comments: ______________________________________
